# A comparison of self-bias measures across cognitive domains

**DOI:** 10.1186/s40359-021-00639-x

**Published:** 2021-09-03

**Authors:** Letizia Amodeo, Jan R. Wiersema, Marcel Brass, Annabel D. Nijhof

**Affiliations:** 1 Department of Experimental Clinical and Health Psychology, Ghent University, Ghent, Belgium; 2 Department of Experimental Psychology, Ghent University, Ghent, Belgium; 3 Berlin School of Mind and Brain/Department of Psychology, Humboldt University of Berlin, Berlin, Germany; 4 EXPLORA, Ghent University, Ghent, Belgium

**Keywords:** Self-bias, Self-related processing, Autism

## Abstract

**Background:**

The ‘self-bias’—i.e., the human proneness to preferentially process self-relevant stimuli—is thought to be important for both self-related and social processing. Previous research operationalized the self-bias using different paradigms, assessing the size of the self-bias *within* a single cognitive domain. Recent studies suggested a reduced self-bias in autism, yet findings are inconsistent. The lack of consensus across existing studies may result from variation in paradigms and cognitive domains tested. Therefore, the primary goal of the current study was to investigate whether self-biases found *across* cognitive domains (i.e., perception, memory, attention) are related or independent. The secondary goal was to explore the relationship between these self-biases and the extent of autistic traits in a neurotypical sample.

**Methods:**

In an online procedure, 99 Dutch-speaking adults performed three self-processing tasks in counterbalanced order—i.e., the shape-label matching task (perception), the trait adjectives task (memory) and the visual search task (attention)—and completed two self-report measures of ASD symptomatology, i.e., AQ-10 and SRS-A. To control for level of familiarity, self-, close other- and famous other-relevant stimuli were included in each task. Repeated measures ANOVAs were conducted for each task, and both frequentist as well as Bayesian analyses were applied to investigate the correlational patterns between self-bias measures.

**Results:**

We observed significant correlations of the self-bias magnitude between memory and attention, as well as attention and perception. However, Bayesian analysis provided only weak support for the latter association. Further, the size of the self-bias was not significantly related across memory and perception. No significant correlation between autistic traits and the self-bias magnitude was found for any of the three tasks, with Bayesian analyses strongly favoring the null hypothesis.

**Conclusions:**

In contrast with the view of a ‘unidimensional’ self-bias, our findings provide evidence for a heterogeneous and multifaceted self consisting of a variety of related and unrelated aspects. None of the self-bias indices were found to relate to autistic traits in our neurotypical sample.

## Background

The ‘self’ is a key concept in the study of human social cognition that has fascinated researchers across many disciplines over the years. In an attempt to clarify the ‘special’ nature of the self [1, 2], several studies demonstrated how the relevance or relatedness to the self can considerably affect information processing [3]. As a matter of fact, individuals are inclined to process stimuli that are self-related in a favored fashion, eliciting a cognitive advantage commonly defined as the ‘egocentric bias’ or ‘self-bias’ [4]. This human predisposition leads individuals to memorize, learn and detect self-relevant stimuli more efficiently [5]. In order to assess to what extent the self-relatedness of a certain stimulus influences an individual’s performance, the self-bias has been operationalized in a wide range of empirical paradigms [6–8]. A multitude of behavioral measures has been employed to investigate the magnitude of self-bias on distinct cognitive domains, such as perception, memory and attention [3, 4]. Nevertheless, it is still unclear which aspects of cognition are mostly affected by the self-bias, and how the self-bias in one domain might relate to the self-bias in another domain. Distinct measures of the self-bias might all draw upon a ‘common’, unitary self-representation; conversely, different aspects of self-representation may underlie distinct measures.

An illustrative example of one of these self-bias measures is the shape-label matching task [9, 10], originally developed to test the effect of novel, transitory self-associations on perception. In this paradigm, participants are first trained to associate geometric shapes with specific labels, indicating either themselves (e.g., ‘you’), a familiar other (e.g., ‘friend’) or an unfamiliar other (e.g., ‘stranger’). Subsequently, participants are presented with either the original shape-label pairings, or new, re-paired associations. The participant’s task is to determine whether the shape matches the label or not on each trial. Results indicate a robust advantage (i.e., faster and more accurate responses) for matching combinations of self-associated stimuli as compared to both familiar and unfamiliar other-associated stimuli. In addition, when the present paradigm was used, self-related associations were found to be less affected by visual degradation than other-related ones (i.e., responses for self-related stimuli were less influenced by contrast reduction [10]), suggesting that self-reference exerts an influence on early-stage, low-level perceptual processing.

In studies exploring the self-bias on memory, there have also been consistent observations of a cognitive advantage in *recalling* self-related over other-related material [11]. This self-bias effect has originally been referred to as the ‘self-reference effect’ (i.e., SRE [12]), which has most often been measured with the well-established trait-adjectives paradigm [12–14]. In this paradigm, participants are asked to judge adjectives in relation to either themselves or to others during a study phase. Another self-processing measure in the memory domain is the ownership task [15], where participants are required to sort objects into self- or other-owned sets during a study phase. In both paradigms, participants later perform a surprise recognition task, which includes the already-seen adjectives/objects as well as distracters, and they are asked to assess whether a specific item has already been presented in the first ‘encoding phase’ or not. Findings from such studies reveal a significantly better memory for objects or trait adjectives that have previously been related to the self as compared to others, and this considerable advantage (i.e., SRE) was found both in typically developing children [16] as well as in adults [15].

A third context in which the preference for self- over other-related information has been discussed is in the attentional domain. Cognitive processing of stimuli such as participants’ own name has been examined using the attentional blink paradigm [17]. This task entails a phenomenon consisting in a decreased ability to detect a second target following a first target in a rapid serial visual presentation (RSVP) stream [18]. When participants are presented with their own name as the second target compared with a close other’s name, the attentional blink is significantly reduced [19]. Similarly, both repetition blindness—a reduced accuracy in reporting the appearance of a stimulus when repeated [20]—and inattentional blindness—an inability to detect an unattended, yet visible stimulus [21]—were found to be diminished for self-referential information [21, 22]. An attentional advantage for self-related material also persistently emerges in the visual search task [23]. Specifically, Yang and colleagues [24] instructed participants to detect either their own, a familiar, or a famous name in an array of distracter names. When searching for their own name, participants showed significantly higher accuracy and faster reaction times than when searching for either familiar or famous names.

It should be noted that thus far, the magnitude of distinct self-bias effects has mostly been investigated in the context of separate studies, making it harder to relate findings from different paradigms to each other. There is a long-established notion that self-reference acts as an ‘integrative hub for information processing’ [3], evenly impacting all cognitive domains. However, this notion has recently been questioned, as it may also be that self-reference affects cognition differently depending on the cognitive domain under investigation: instead of unidimensional and homogeneous, its effects may be diversified and multifaceted. To address this question, a direct comparison of the different self-biases within one and the same sample is required. To our knowledge, only one study directly compared the self-bias effects across cognitive domains within the same sample: Nijhof and colleagues [19] administered two self-processing measures—respectively in the context of attention and perception—within the same experimental procedure, and found no evidence for a common mechanism that underlies the self-biases across these domains. Such findings endorse a view of the self-bias as a heterogeneous phenomenon that does not draw upon a shared, underlying self-representation.

Further support for a non-unitary impact of the self-bias on cognition comes from the body of literature that has investigated autism-related alterations in self-bias effects. Regardless of the cognitive domain under study, it is believed that the self-bias fosters social competence, as a stronger sense of self is thought to help one build a better model of the social world [5, 25, 26]. An altered self-bias has in turn been argued to lead to social impairments by impeding the understanding of other people’s emotions, intentions or beliefs [19, 27, 28]. This led to a growing number of studies comparing self-processing between individuals with and without an autism diagnosis, as well studies investigating the relationship between autism characteristics/traits in neurotypical samples. This latter approach has recently been applied more frequently and is based on the acknowledgement that despite autism being considered a clinical condition, autistic characteristics or traits are continuously distributed in the general population [29, 30]. Indeed, previous studies in neurotypicals have shown the potential value of correlational approaches exploring the relationship between autistic traits and task performance in neurotypicals for relevant insights about autism [30–33]. Current findings from both clinical and non-clinical studies are however inconsistent, with autism-related reduced self-bias effects found for some of the aforementioned paradigms, but not for others. For instance, the self-bias on the shape-label matching task showed no significant association with autistic traits in neurotypicals [19], and its magnitude did not differ between autistic adults and matched controls [29]. As for the attentional domain, Nijhof and colleagues [19] observed no significant correlation between autistic traits and the self-bias magnitude using the attentional blink paradigm in a neurotypical sample. In contrast, in the memory domain, the self-bias effect was found to be absent or significantly reduced in autistic children [34, 35], adolescents [27] and adults [14, 36, 37]. Nevertheless, more recent research challenged these results, suggesting no significant relation between the self-bias magnitude in the memory domain and autistic traits, in both neurotypicals as well as individuals with autism [38]. In summary, despite some evidence pointing out autism-related altered self-processing, it is clear that findings are still mixed. While the inconsistency may partly be due to the inclusion of a range of different samples (neurotypicals with different levels of autistic traits, individuals with autism, children, adults) in the conducted studies, the inconsistency of findings may further point to a non-unitary impact of the self-bias on cognition.

When confronted with such conflicting results, one could indeed argue that the inconsistency of the current findings may relate to the lack of convergence with regard to the type of self-representations under investigation [29]. It has been suggested that early-stage processing of self-referential material (e.g., tagging an item as ‘self-related’) may be intact in autism, while late-stage self-referential processing (e.g., assessing whether an adjective applies to the self or not) may be impaired. This distinction between early- and late-stage processing of self-related information resembles James’ conceptualization of first and second-order self-representations [39]: stimuli can be labelled as merely self-related (first-order processing, e.g. “Self = circle”) or can in turn be processed as the object of one’s own thought (second-order processing, e.g., “Does the word ‘intelligent’ describe me?”). In this respect, the impact of self-relevance might change considerably depending on the cognitive process it affects.

To date, the aforementioned investigation conducted by Nijhof and colleagues [19] constitutes the only attempt to administer distinct self-bias measures across cognitive domains in a common sample of participants. Moreover, the way different measures of self-processing relate to each other needs additional investigation in both neurotypical and autistic populations [5]. Research on cognitive functioning in typically developed individuals can shed light on relevant features characterizing how this cognitive functioning might be altered in a range of psychiatric, neurological or neurodevelopmental conditions. Specifically, a more exhaustive knowledge of whether the self-bias effects can be explained by one unitary or separate mechanisms in neurotypicals can potentially improve our understanding of how these effects were sometimes—although not consistently—found to be reduced in autism or in those with more autistic traits. Furthermore, overall conclusions on the impact of self-relatedness on cognition have generally been drawn on the basis of relatively small samples, that were possibly lacking sufficient statistical power [15, 22, 36]. Under-powered studies risk producing a larger quantity of false negatives than high-powered studies [40], and tend to report statistically significant findings which actually have a relatively low likelihood of reflecting authentic effects [41]. Using adequately powered samples to study self-processing and its relation to autistic traits has therefore become imperative.

In light of the above, the main aim of the present study is to investigate self-bias effects across distinct cognitive domains, by administering and comparing three self-processing measures, i.e., the shape-label matching task (perceptual domain), the trait adjectives task (memory domain), and the visual search task (attentional domain), within the same experimental procedure. The current investigation has two foremost goals. The primary goal is to explore whether self-biases in attention, memory and perception are related. That is: do they result from a shared mechanism across cognitive domains, or instead reflect unrelated effects (as was found for the self-biases on the shape-label matching task and the attentional blink task [19])? The secondary goal is to investigate the associations between different self-processing measures and questionnaires assessing autistic traits in a neurotypical sample to provide a better understanding of how self-bias effects might be linked to autism symptomatology. Although the only previous study on the comparison of self-biases across different domains conducted by Nijhof and colleagues [19] reported the lack of a shared mechanism underlying these effects, our investigation employs distinct self-processing paradigms from those used in the aforementioned study. We use paradigms consisting both of higher-order self-processing (trait adjectives task), attentional self-processing (visual search task), and low-level perceptual self-processing (shape-label matching task). As a result, both converging and diverging self-bias effects may be expected. Furthermore, a dimensional approach will be applied exploring possible associations between the magnitude of different self-bias effects and autistic traits. As a complementary approach to clinical research in autistic individuals, studying autism-related traits in neurotypicals is indeed considered to be an effective method for investigating relevant features of autism which has been adopted successfully by previous studies (see, e.g., [30–33]). Despite reports of a link between reduced self-bias and autism, findings are not consistent: investigating self-bias across different domains within the same sample may provide more clarity.

## Materials and methods

### Participants

Based on the assumption that small-sized correlations would emerge by comparing the self-bias measures across the different tasks and to the self-report measures,^1^ our goal was to test a minimum of 97 participants, as this would provide 80% power in detecting relatively small-sized correlations of r = 0.25 at α = 0.05. However, after initially noticing a higher-than-average drop-out rate (probably due to the fact that the study was conducted in an online environment), we decided to recruit a larger number of participants than our sample size estimate. In order to obtain usable data from at least 97 individuals, 132 Dutch-speaking participants were recruited via online advertisements. Twenty-seven participants did not report their own name or the name of their close other in the correct format, i.e., all capitals for the shape-label matching task and only first letter as a capital for the visual search task (in line with previous studies; 19,24]. As these typing errors might have influenced their performance (e.g., participants might have shown faster reaction times when searching for a name typed in capitals in the visual search task, since such a name could result as a more salient stimulus in an array of names typed with the first letter in uppercase only), these data were excluded from further analyses. No participant reported any diagnosis of neurological or mental health difficulties. Six participants were removed from the present sample for having 60% or lower accuracy in the matching phase of the shape-label matching task. As a result, the ultimate sample consisted of 99 participants (23 male, mean age: 23.7 ± 5.1 years). Most individuals were right-handed (94.9%), Caucasian (96.9%) and had Dutch as their native language (96.0%). All participants gave informed consent prior to the study and were financially compensated for their participation. The study was approved by the ethical committee of the Faculty of Psychology and Educational Sciences at Ghent University (approval code 2020/08).

### Procedure

The whole experimental procedure was programmed to be carried out online using Gorilla Experiment Builder [42]. Participants were required to perform the three self-processing tasks (i.e., the shape-label matching, the trait adjectives, and the visual search tasks), and subsequently completed three questionnaires (i.e., the 10-item Autism Spectrum Quotient, the Social Responsiveness Scale—Adult version and the Self-Consciousness Scale-Revised). The order of both tasks and questionnaires was counterbalanced across participants. To control for level of familiarity, either self-, close other-, or famous other-relevant stimuli were presented in each task. Before starting any task, participants were shown the following instruction screen: ‘In this experiment, you will be regularly asked to think about a ‘friend’. By this word, we mean someone who knows you well and who is close to you. You can decide to think about your partner, one of your best friends, a family member, … The most important thing is that you know that person well. It is important that you think about the same person every time you are presented with the word ‘friend’, across all the three tasks you will perform.’ Before performing the shape-label matching and the visual search tasks, participants were instructed to provide their own name and their close other’s name. In line with previous research [19, 24], they were instructed to use all capitals when typing the names for the shape-label matching task, whereas they had to type only the first letter in capital when entering the names for the visual search task.

### Tasks

#### Shape-label matching task

##### Apparatus

The present task is based on the original paradigm developed by Sui and colleagues [10] and on the online-adapted procedure implemented by Nijhof and colleagues [19]. The task stimuli consisted of three uniformly white colored, 3.3° × 3.3° sized geometric shapes (circle, square, triangle), and three labels (white ink, capitalized 40-point Arial). To control for level of familiarity and to allow comparisons with any found self-biases effect on the trait adjectives and the visual search tasks, participants were presented with their own first name, the first name of their close other (e.g., their best friend), and the first name of a famous other (i.e., Harry, mentioned to be Harry Potter) as opposed to the labels used in previous research (‘you’, ‘friend’, ‘stranger’; [10, 43]).

##### Association phase

At the beginning of the task, the following instructions were displayed on the screen, individualized per participant: ‘In this part of the experiment, you will learn to associate shapes with labels: the ‘Circle/Square/Triangle’ with ‘Name 1’, the ‘Circle/Square/Triangle’ with ‘Name 2’, and the ‘Circle/Square/Triangle’ with ‘Name 3’. In each trial, you will be asked to determine which label matches the shape displayed on the screen. Please press left for ‘Name 1’, down for ‘Name 2’, and right for ‘Name 3’.’ At the beginning of each trial, a fixation cross was centrally presented for 2000 ms. One of the three shapes and the three labels appeared concomitantly on a light grey background for 1000 ms. The shape was displayed above the fixation cross, whereas the labels were presented on the left, middle and right lower half of the screen. The shape-label association as well as the location in which the labels appeared on the screen were counterbalanced across participants. Subsequently, a fixation cross was displayed for 2000 ms, or until participants' response (provided by left, down or right arrow). Finally, feedback was presented for 500 ms: a green check mark was displayed below the fixation in case participants answered correctly within the 2000 ms time limit; whereas a red cross was displayed in the same location if they answered incorrectly or if they did not provide any response within the time limit. Participants initially performed six practice trials, followed by the actual task, which instead consisted of 24 trials.

##### Matching phase

All the stimuli in the following, matching phase corresponded to the ones presented in the association phase. An initial fixation cross was centrally displayed on screen for 500 ms. Subsequently, a shape was displayed above the fixation cross and a label below it, both appearing concomitantly for 150 ms, on a light grey background. Stimulus presentation was followed by an inter-trial interval of 1000 ms. Participants were asked to respond to a matching shape-label pair with either the left or the right arrow key, and with the opposite arrow key for a mismatching pair. Left and right arrow key order was counterbalanced between participants. This task phase included 12 practice and 360 test trials, divided into three blocks of 120 trials each. Within each block, 20 matching and 20 non-matching combinations were randomly presented for each of the three label conditions. Participants received feedback on each trial for 500 ms, as in the association phase.

#### Trait adjectives task

##### Apparatus

The present task is an online-adapted version of a depth-of-processing paradigm used in previous research to elicit the self-referential effect [12–14]. The stimuli comprised of a list of 240 Dutch trait adjectives, divided across five conditions (30 items in each of four conditions in the encoding phase, 120 novel items in the recognition phase), with the five lists matched for number of syllables, frequency and valence of the adjectives (see Table 1). The association between trait adjectives and condition was counterbalanced across participants, except for the list of novel items.

**Table 1:** Mean frequency and number of syllables for the adjectives lists of the trait adjectives task

	Syllables	Frequency
Distracters’ list	2.87 (1.03)	11.52 (31.42)
List 1	2.97 (0.89)	13.81 (31.78)
List 2	3.13 (0.78)	7.93 (14.75)
List 3	2.93 (0.98)	14.02 (36.50)
List 4	3.13 (0.86)	6.70 (10.98)

Each list included an equal percentage of positive and negative adjectives. Standard deviations are reported in brackets

##### Encoding phase

During the encoding phase, trait adjectives were centrally displayed on the screen, and participants had to make judgments about them in one of four ways. In the Self condition, participants judged how descriptive a specific adjective was of themselves. In the Close Other condition, the trait adjective was in turn judged on how descriptive it was of their best friend. In the Famous Other condition, participants provided a judgment on whether the adjective was descriptive of Harry Potter. Participants were asked to rate to what extent these words were descriptive of any of these persons on a 6-point scale, in which 1 indicated “Not at all descriptive” and 6 indicated “Very descriptive”. In contrast, in the non-social control condition, participants were asked to determine how many syllables each adjective contained, again using a 6-point scale (1–6 syllables). Labels specifying each condition (i.e., ‘Self’, ‘Friend’, ‘Harry Potter’, ‘Syllable’) were displayed on the top of the screen. Each participant performed 120 trials (30 trials per condition) presented in a pseudorandomized order.

##### Recognition test phase

After completing the encoding phase, participants were presented with a surprise memory recognition test. All 120 trait adjectives from the encoding phase and 120 new distracter trait adjectives were presented in pseudorandomized order. In this task, participants were asked to rate their confidence in recalling the word displayed at the center of the screen. Specifically, they had to judge to what extent a certain adjective was ‘old’ (i.e., seen during the encoding phase) or ‘new’ (i.e., not seen during the encoding phase), using a 6-point scale (1 = ‘I think this word is new’, 3 = ‘I think this word is new, but I am kind of unsure’, 4 = ‘I think this word is old, but I am kind of unsure’, 6 = ‘I think this word is old’).

#### Visual search task

##### Apparatus

This task is an online-adaptation based on the study of Yang and colleagues [24]. The apparatus consisted of Dutch first names which were visually displayed on the screen. The stimuli included participant’s own name, a close other’s name (e. g., best friend’s name), a famous other’s name (i.e., Harry (mentioned to be Harry Potter)), and a list of 48 names, which were randomly selected from a list of commonly used first names in the Flemish Region provided by the official Belgian statistical institution, Statbel [44]. The latter names were used as distractor items in the visual search task. The distractor names were matched for word length, number of syllables, and gender.

##### Visual search task

The task comprised three blocks, each consisting of 96 trials, in which participants were instructed to search for one specific target: their own name, their best friend’s name, or the famous other’s name. The order of the three blocks was counterbalanced across participants. At the beginning of each block, participants were informed about which target to search for. Within each block, the target name was only present on half of the trials. In each trial, five or six distractor names were selected randomly from the total list of 48 common names. Participants were asked to judge as quickly and accurately as possible whether the target name was present. They were also instructed to use the index finger of their dominant hand to press the spacebar key in response to the target name. In case the target was absent, participants were asked not to press any key, and just wait for the next trial. At the start of each trial, a fixation cross was displayed for 500 ms, followed by an array of six names. The names were evenly presented around a central point and formed a virtual circle of 13° visual angle, based on a viewing distance of 60 cm. The stimuli were displayed on the screen for 2000 ms or until participants’ response. Subsequently, a blank screen appeared for 1000 ms. The frequencies with which each target name was presented in one of the six possible locations were balanced across participants.

### Questionnaires

To explore possible associations between any found self-bias effects and autism symptomatology, all participants completed the *10-item Autism Spectrum Quotient* (AQ-10 [45]), a brief self-report questionnaire which measures autistic-like traits in neurotypical individuals. In addition, they filled out the *Social Responsiveness Scale*—*Adult version* (SRS-A [46]), a 64-items instrument measuring autistic symptom severity, that can be used as a screener in the general population or as an aid to clinical assessment. In both measures, each item is rated on a 4-point scale. In the AQ-10, participants provided a rating for each item between 1 and 4, where 1 indicated they ‘totally disagree’ with the content of the item and 4 indicated ‘totally agree’. In the SRS-A, participants rated each item on the extent to which it applied to them in the last 6 months, using a scale between 1 and 4, in which 1 meant ‘not true’ and 4 meant ‘almost always true’. Participants also completed the *Self-Consciousness Scale-Revised* (SCS-R [47]), a self-report measure assessing private and public self-consciousness. Participants provided a rating on how descriptive each item was with reference to themselves using a 4-point scale, in which 1 indicated ‘not like me at all’ and 4 indicated ‘a lot like me’.

### Analysis

The statistical analyses were conducted using IBM SPSS, version 25 [48]. Partial eta squared (η_p_^2^) values and Cohen's d are reported as measures of effect size for analyses of variance (ANOVAs) and t-tests, respectively. The F-values resulting from the ANOVAs are reported sphericity-assumed. However, if the assumption of sphericity was violated, a Greenhouse–Geisser correction was applied, and the corrected *p*-values were reported.

Following previous research using the shape-label matching task and visual search task [19, 24, 29] self-bias effects were based on reaction time (RT), which has been shown the most reliable measure to index self-bias effects in these paradigms. However, for completeness, accuracy results are reported for these tasks as well. In the trait adjectives task, the self-bias index was based on participants’ average rating on the 6-point scale during the recognition phase (see below). Instead of using d’, we used the 6-point scale rating as dependent variable, in order to more accurately capture the variability in participants’ responses between conditions, and to obtain a more ‘nuanced’ estimate of the self-bias in this task.

Based on the analysis strategy of Sui and colleagues [10], data from the shape-label matching task were analyzed by performing a 2 (Matching/Non-Matching) × 3 (Name) repeated-measures analysis of variance (ANOVA), for both RT on correct trials, and accuracy. In the present task, the self-bias effect is commonly observed in the matching trials. Hence, follow-up analyses for matching and non-matching pairs were planned separately. Outliers were removed on an individual basis: responses shorter than 100 ms and/or three standard deviations (SDs) above or below each participant’s mean were excluded from further analysis, eliminating 0.6% of the trials overall.

For the visual search task, the RT analysis strategy was based on the study of Yang and colleagues [24]. For each participant, RTs faster than 100 ms and/or exceeding three standard deviations (SDs) above or below the mean were removed, eliminating less than 1.1% of the trials overall. Subsequently, a repeated measures ANOVA with three within subject levels (Own name, Close Other name, Famous Other name) was performed. The hit rate was computed on an individual basis as the number of trials in which the participant correctly responded out of the total number of trials where the target name was present. The percentage of false alarms was in turn individually calculated based on the number of trials in which the participant erroneously responded out of the total number of trials in which the target was absent. Hit rates and false alarms percentage were computed separately for each condition.

With regard to the trait adjectives task, we ran a repeated measures ANOVA with five within subject levels (Self, Close Other, Famous Other, Syllable, Distracter). Our dependent variable consisted in participants’ ratings on the 6-point scale, in which higher values indicated greater confidence in recognizing the trait adjective as an ‘old’, already-seen word, whereas lower values indicated greater confidence in identifying the trait adjective as a new item.

Correlational analyses were performed on self-bias effects across the three tasks, as well as between the three self-bias measures and participants' AQ-10, SRS-A and SCS-R scores. In line with previous research [19, 24, 29], the self-bias effects on the visual search task and on the matching trials in the shape-label matching task were calculated on the basis of participants’ mean RT (rather than accuracy scores). On both tasks, the self-bias effect was operationalized as the participant’s average RT difference between the Self and Close Other conditions, divided by the sum of the average Self and Close Other RT differences (i.e., (mean RT Self—mean RT Close Other) / (mean RT Self + mean RT Close Other)), in order to eliminate the confound of interindividual mean speed differences. In the trait adjectives task, the self-bias was measured as the participant’s average 6-point rating difference between the Self and Close Other conditions. In addition, Shapiro–Wilk tests were conducted to assess whether the self-bias measures as well as the questionnaires scores had normal distributions. No significant departures from normality were shown for the distributions of the self-bias measures (separately calculated for each task: trait adjectives task, W(99) = 0.984,* p* = 0.293; visual search task, (W(99) = 0.992,* p* = 0.847; shape-label matching task, W(99) = 0.982,* p* = 0.213), as well as for the distribution of the SCS-R scores (W(99) = 0.986,* p* = 0.396). On the contrary, both the AQ-10 and the SRS-A scores showed major departures from the normal distribution (W(99) = 0.922,* p* < 0.001; W(99) = 0.938,* p* < 0.001, respectively). To further conduct the correlational analyses, parametric tests (i.e., Pearson’s r) were performed for normally distributed data, whereas non-parametric tests (i.e., Kendall’s tau-b) were used for data showing significant departures from normality.

Finally, drawing conclusions based on both the frequentist and the Bayesian approaches is considered crucial to deal with the ever-increasing complexity of current research questions [49]. For this reason, Bayesian correlational analyses were additionally conducted using JASP [50]. Within this framework, a Bayes Factor is computed as the ratio of the likelihood of one specific hypothesis to the likelihood of the other, and represents the weight of evidence in favor of the null (r = 0) and alternative hypotheses (r ≠ 0). Larger values of the Bayes Factor (BF_10_) indicate stronger evidence in favor of the alternative hypothesis (H1) compared to the null hypothesis (H0). When this value approaches 1, it indicates that H0 and H1 are equally probable, whereas values below 1 provide greater evidence in support of H0. Based on previous research investigating correlations between self-bias measures [19] and in order to provide an appropriate estimate of H1 effect size, we entered the value of 0.5 as the stretched beta prior width.

## Results

### Shape-label matching task

To analyze RT data, we conducted a 2 (Trials: Matching vs. Mismatching) × 3 (Name: Self vs. Close Other vs. Famous Other) repeated-measures ANOVA. Analysis of RT data revealed a significant main effect of Name (F(2, 196) = 59.42, *p* < 0.001, η_p_^2^ = 0.38), with faster responses for pairings which involved the Self-label compared to either the Close Other or the Famous Other. The effect of Trials (Matching/Non-Matching) was also found to be significant (F(1, 98) = 449.85,* p* < 0.001, η_p_^2^ = 0.82): participants were significantly faster in responding to matching pairs than mismatching pairs. Moreover, the interaction effect between Name and Trials was significant (F(2, 196) = 26.78,* p* < 0.001, η_p_^2^ = 0.21). As a consequence, we performed planned comparisons for the effect of Name on matching and non-matching trials separately (see Table 2). For the matching trials, results indicated a significant Name effect (F(2, 196) = 59.30,* p* < 0.001, η_p_^2^ = 0.38), with planned comparisons showing significantly faster responses with pairings involving the Self compared to the Close Other (t(97) = 5.91,* p* < 0.001, d = 0.52) as well as to the Famous Other (t(97) = 10.75,* p* < 0.001, d = 0.86). Additionally, RTs were significantly faster in the Close Other condition compared to the Famous Other condition (t(97) = 4.98,* p* < 0.001, d = 0.38). With regard to the mismatching trials, the effect of Name was also found to be significant (F(2,196) = 16.13,* p* < 0.001, η_p_^2^ = 0.14). Planned comparisons indicated significant differences between the Self and the Close Other conditions (t(97) = 5.99,* p* < 0.001, d = 0.33), as well as between the Self and the Famous Other conditions (t(97) = 3.38,* p* = 0.003, d = 0.22): faster RTs were observed with mismatching pairings involving the Self-label compared to both the Close Other and the Famous Other. However, RTs in the Close Other condition did not significantly differ from RTs in the Famous Other condition (t(97) = 1.98,* p* = 0.153, d = 0.12).

**Table 2:** Mean accuracy and reaction times (ms) for each condition in the shape-label matching task

	Accuracy	RTs (ms)
*Match*		
Famous other	.79 (.12)	710.65 (64.00)
Close other	.85 (.11)	687.62 (56.15)
Self	.88 (.09)	658.16 (57.90)
*Mismatch*		
Famous other	.85 (.11)	748.55 (56.22)
Close other	.83 (.10)	755.23 (58.42)
Self	.82 (.11)	735.86 (58.50)

Standard deviations are reported in brackets

As for the analysis of accuracy data, we performed a 2 (Trials: Matching vs. Mismatching) × 3 (Name: Self vs. Close Other vs. Famous Other) repeated-measures ANOVA. Results showed a non-significant main effect of Trials (F(1, 98) = 1.31,* p* = 0.256, η_p_^2^ = 0.01), yet a significant main effect of Name (F(2, 196) = 7.77,* p* = 0.001, η_p_^2^ = 0.07), with participants responding more accurately when presented with associations involving a geometric shape and the Own name compared to either Close Other’s or Famous Other’s names. Furthermore, the interaction effect between Name and Trials was significant (F(2, 196) = 27.72,* p* < 0.001, η_p_^2^ = 0.22). Data from matching and mismatching trials were analyzed separately (see Table 2): for matching pairs, a significant effect of Name was found (F(2, 196) = 25.80,* p* < 0.001, η_p_^2^ = 0.21), with higher accuracy on the Self condition compared to both the Close Other (t(97) = 3.10,* p* = 0.008, d = 0.34) and the Famous Other (t(97) = 6.89,* p* < 0.001, d = 0.84) conditions. Additionally, participants’ accuracy level in the Close Other condition was significantly increased compared to the Famous Other condition (t(97) = 4.07,* p* < 0.001, d = 0.49). With regard to non-matching trials, the main effect of Name on participants’ accuracy was also significant (F(2, 196) = 5.65,* p* = 0.005, η_p_^2^ = 0.06). Accuracy level on the Self condition was found to be significantly lower than Famous Other condition (t(97) = 2.94,* p* = 0.012, d = 0.27). No significant difference in accuracy was found between Self and Close Other conditions (t (97) = 1.06,* p* = 0.871, d = 0.08), nor between Famous Other and Close Other conditions (t(97) = 2.38,* p* = 0.058, d = 0.21).

### Trait adjectives task

A repeated measures ANOVA with five within-subject levels of Condition (Self, Close Other, Famous Other, Syllable, Distracter) was conducted on the trait-adjectives task data. Participants’ mean responses in the recognition phase for each condition are displayed in Table 3. Results of the ANOVA revealed a significant effect of Condition (F(4, 392) = 326.10,* p* < 0.001, η_p_^2^ = 0.77). Planned comparisons indicated an enhanced advantage in the recognition of self-related adjectives compared to all the other conditions. Participants provided significantly higher ratings on a 6-point scale during the recognition phase when judging items that had been previously processed with reference to the Self compared to either Close Other (t(95) = 5.15,* p* < 0.001, d = 0.37) or the Famous Other (t(95) = 12.17,* p* < 0.001, d = 1.25). In addition, participants better recognized trait adjectives processed under the Self condition compared to the Syllable condition (t(95) = 16.15,* p* < 0.001, d = 2.00). The difference between the Self and the Distracter conditions was also found to be significant (t(95) = 24.14,* p* < 0.001, d = 3.91), revealing greater recognition for self-related items compared to new, distracting ones. Additionally, individual ratings significantly increased during the recognition phase with items that had been previously processed in relation to the Close Other compared to either the Famous Other (t(95) = 8.14,* p* < 0.001, d = 0.85) or the Syllable condition (t(95) = 13.46,* p* < 0.001, d = 1.57). Finally, participants also showed a better recognition for adjectives processed in the Close Other condition than for Distracter adjectives (t(95) = 21.04,* p* < 0.001, d = 3.42).

**Table 3:** Mean response for each condition during the recognition phase of the trait adjectives task

	Response
*Condition*	
Distracter	2.19 (.62)
Syllable	3.40 (.59)
Famous other	3.84 (.63)
Close other	4.38 (.66)
Self	4.62 (.63)

Participants provided a rating on a 6-point scale that ranged from 1 (i.e., ‘Definitely new’) to 6 (i.e., ‘Definitely old’). Standard deviations are reported in brackets

### Visual search task

To analyze participants’ RTs, a repeated measures ANOVA with three within-subject levels of Condition (Own name, Close Other’s name, Famous Other’s name) was conducted. The effect of Condition was significant, revealing different search speeds for the three target names (F(2, 196) = 49.09,* p* < 0.001, η_p_^2^ = 0.33): the mean RT for participant’s Own name was shorter than for either the Close Other’s name or Famous Other’s name (see Table 4). Planned comparisons showed that participants were significantly faster in searching for their Own name than for either the Close Other’s name (t(97) = 5.67,* p* < 0.001, d = 0.64) or the Famous Other’s name (t(97) = 11.01,* p* < 0.001, d = 1.00). Moreover, there was a significantly higher detection speed when searching for the Close Other’s name as a target compared to the Famous Other’s name (t(97) = 3.27,* p* = 0.004, d = 0.29).

**Table 4:** Mean hit rates, false alarm rates and reaction times (ms) in the visual search task

	Hit rate	False alarm rate	RTs (ms)
*Condition*			
Famous other’s name	.96 (.03)	.02 (.02)	865.66 (133.17)
Close other’s name	.98 (.03)	.02 (.05)	823.28 (156.48)
Own name	.99 (.02)	.03 (.03)	728.57 (139.81)

Standard deviations are reported in brackets

Accuracy data were analyzed by performing a repeated measures ANOVA with three within-subject levels of Condition (Own name, Close Other’s name, Famous Other’s name). Results indicated a significant main effect of Condition (F(2, 196) = 23.44,* p* < 0.001, η_p_^2^ = 0.19), suggesting accuracy differed depending on the target name participants were given. Planned comparisons further revealed that participants were significantly more accurate in searching for their Own name than for the Famous Other’s name (t(97) = 6.40,* p* < 0.001, d = 0.95). Under the Close Other’s condition, accuracy level in the target name search was significantly higher compared to Famous Other’s condition (t(97) = 4.32,* p* < 0.001, d = 0.63). However, participants’ accuracy did not significantly differ when searching for their Own name compared to the Close Other’s name (t(97) = 2.31,* p* = 0.068, d = 0.32). A similar repeated measures ANOVA was conducted on false alarms, indicating no significant effect of target identity on false alarm rate (F(2, 196) = 2.76,* p* = 0.082, η_p_^2^ = 0.03).

In summary, a consistent advantage of the self-related condition over the close other-related condition was observed across all the three paradigms we used to operationalize the self-bias effect in distinct domains of cognition: the shape-label matching task (perception), the visual search task (attention), and the trait adjectives task (memory). Similarly, a significant—although less marked—advantage of the close other-related condition over the famous other-related condition was consistently found across all tasks.

### Comparisons across tasks

**Fig. 1:**
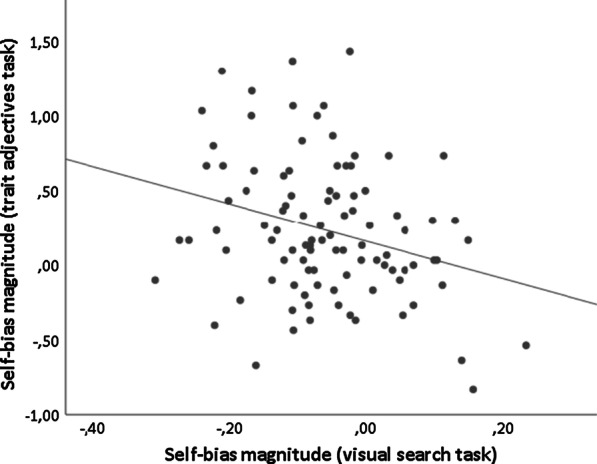
Correlation between the magnitude of the self-bias effect found in the trait adjectives task and the magnitude of the self-bias effect found in the visual search task (r(99) = − .29,* p* = .004; BF_10_ = 10.163)

**Fig. 2:**
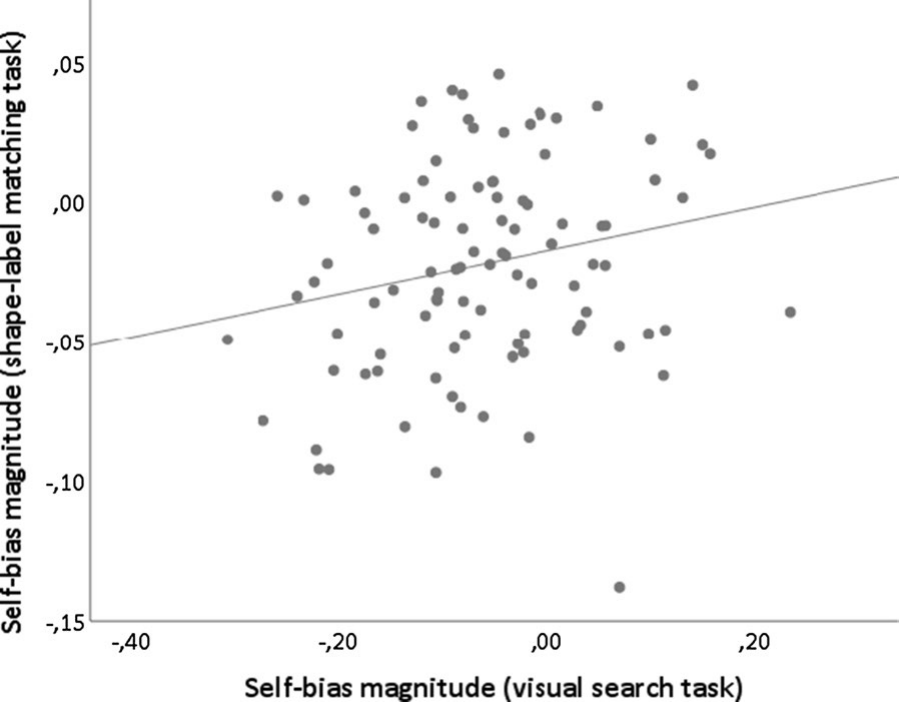
Correlation between the magnitude of the self-bias effect found in the shape-label matching task and the magnitude of the self-bias effect found in the visual search task (r(99) = .22,* p* = .030; BF_10_ = 1.822)

**Fig. 3:**
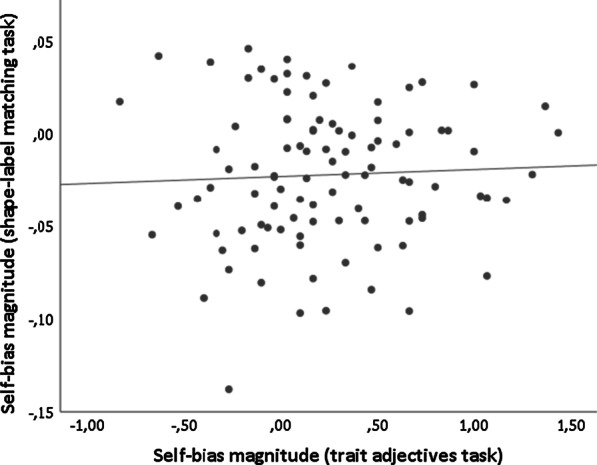
Correlation between the magnitude of the self-bias effect found in the trait adjectives task and the magnitude of the self-bias effect found in the shape-label matching task (r(99) = .05,* p* = .649; BF_10_ = 0.206)

Test statistics, significance levels and Bayes Factors from correlational analyses are reported in Table 5. Results showed that the self-bias magnitude on the trait adjectives task was significantly associated with the self-bias magnitude on the visual search task, r(99) = − 0.29,* p* = 0.004, BF_10_ = 10.163 (see Fig. 1). Thus, higher scores on the 6-point scale for self-referential than other-referential words in the recognition phase of the trait adjectives task significantly correlate with faster RTs in detecting own name compared other-related names (i.e., a negative correlation actually indicates a positive relation between the self-bias magnitude across the tasks). Substantial evidence was provided in favor of the alternative hypothesis (r ≠ 0) compared to the null hypothesis (r = 0) for the correlation between the self-bias effects on the trait adjectives and the visual search tasks according to Bayesian analysis. In addition, the size of the self-bias observed in the visual search task was significantly related to the magnitude of the self-bias found in the shape-label matching task (r(99) = 0.22,* p* = 0.030, see Fig. 2). That is, shorter RTs in response to self-related compared to other-related matching pairs in the shape-label matching task were found to be significantly correlated with faster responses in searching for one’s own name compared to the names of others in the visual search task. However, additional Bayesian analysis provided only weak, anecdotal evidence in favor of the alternative hypothesis for the correlation between the self-bias effects on the visual search and the shape-label matching tasks (BF_10_ = 1.822). Moreover, the size of the self-bias was not significantly related across the trait adjectives and the shape-label matching tasks (r(99) = 0.05,* p* = 0.649, see Fig. 3), and Bayesian analysis further confirmed this lack of significance (BF_10_ = 0.206).

**Table 5:** Correlational analyses between self-bias magnitudes and AQ-10, SRS-A and SCS-R scores

Variable	Statistics	Bias (TA)	Bias (SLM)	Bias (VS)	AQ-10	SRS-A	SCS-R
Bias (TA)	r	–					
	*p*	–					
	BF_10_	–					
Bias (SLM)	r	0.046	–				
	*p*	0.649	–				
	BF_10_	0.206	–				
Bias (VS)	r	− 0.287	0.218	–			
	*p*	0.004**	0.030*	–			
	BF_10_	10.163*	1.822	–			
AQ-10	τb	0.091	0.074	0.018	–		
	*p*	0.217	0.309	0.805	–		
	BF_10_	0.458	0.345	0.201	–		
SRS-A	τb	0.038	− 0.048	0.074	0.340	–	
	*p*	0.586	0.487	0.282	< .001***	–	
	BF_10_	0.226	0.247	0.345	31,469.77***	–	
SCS-R	r	0.141	0.129	− 0.080	–	–	–
	τb	–	–	–	0.144	0.208	–
	*p*	0.164	0.204	0.429	0.053	0.003**	–
	BF_10_	0.326	0.278	0.171	1.174	13.231*	–

Self-bias magnitude in trait adjectives task = ‘Bias (TA)’; self-bias magnitude in shape-label matching task = ‘Bias (SLM)’; self-bias magnitude in visual search task = ‘Bias (VS)’. Pearson’s r (r), *p*-values (*p*), Kendall’s tau (τb) and Bayes Factor (BF_10_; stretched beta prior width = 0.5) are reported

^*^p < .05; **p < .01; ***p < .001; *BF_10_ > 10; **BF_10_ > 30; ***BF_10_ > 100

### Associations between self-bias measures and questionnaire scores

In addition, the magnitude of the self-bias effect was not related to SCS-R scores across any of the three tasks (trait adjectives task: r(99) = 0.14,* p* = 0.164; shape-label matching task: r(99) = 0.13,* p* = 0.204; visual search task: r(99) = -0.08,* p* = 0.429), and Bayesian analyses provided evidence favoring the null hypothesis for all the correlations (trait adjectives task: BF_10_ = 0.475; shape-label matching task: BF_10_ = 0.407; visual search task: BF_10_ = 0.252). Furthermore, the size of the self-bias effect in any of the tasks was found to be unrelated to the SRS-A total score (trait adjectives task: τb(99) = 0.04,* p* = 0.586; shape-label matching task: τb(99) = − 0.05,* p* = 0.487; visual search task: τb(99) = 0.07,* p* = 0.282), with Bayesian analyses providing evidence in support of the null hypothesis across all the domains (trait adjectives task: BF_10_ = 0.226; shape-label matching task: BF_10_ = 0.247; visual search task: BF_10_ = 0.345). Finally, no significant correlation between autism symptomatology—as assessed by the AQ-10—and the size of the self-bias effect was found across any of the three self-processing measures (trait adjectives task: τb(99) = 0.09,* p* = 0.217; shape-label matching task: τb(99) = 0.07,* p* = 0.309; visual search task: τb(99) = 0.02,* p* = 0.805). The lack of relationship between AQ-10 scores and self-bias effects was also confirmed by further Bayesian analyses (trait adjectives task: BF_10_ = 0.458; shape-label matching task: BF_10_ = 0.345; visual search task: BF_10_ = 0.201).

## Discussion

The primary goal of the current study was to further explore the human tendency to preferentially process self-relevant stimuli, by comparing the self-bias effects found across the perceptual, the attentional and the memory domain. By testing an adequately-powered sample of neurotypicals, we aimed to investigate whether the cognitive advantages for self-related information observed in three self-processing paradigms—i.e., the shape-label matching task (perception), trait adjectives task (memory), and visual search task (attention)—can be explained by a shared underlying mechanism (unidimensional view) or rather reflect distinct self-related processes. Our study provided further proof for a consistent self-bias effect across all the domains of cognition investigated. Most importantly, when comparing distinct measures of the self-bias across cognitive domains, the correlational patterns differed based on the domains involved, challenging a unitary view of the self-bias. No evidence was found for an association between self-reported autistic traits and the magnitude of the self-bias effect on either of the three tasks.

Our findings of a specific preference for self-related stimuli across all the three tasks were in line with previous research, and could not be accounted for by a general familiarity effect (since self-related conditions were compared to both familiar and non-familiar other-related conditions in all three paradigms [10, 14, 24]). In addition, results from correlational analyses showed a reliable association between the self-bias effects on the visual search task (i.e., attentional domain) and the trait adjectives task (i.e., memory domain), with Bayesian analyses strongly favoring the alternative hypothesis for this correlation. The self-bias in the shape-label matching task (i.e., perceptual domain) was found to be significantly related to the one in the visual search task (attentional domain), however Bayesian analyses provided only anecdotal support for this link. No significant association was observed when comparing the self-bias effects found in the trait adjectives task and in the shape-label matching task, with Bayesian analysis strongly supporting the null hypothesis of no correlation between these measures.

These findings challenge the unidimensional standpoint on the self-bias, which conceptualizes self-reference as an associative core for information processing, consistently affecting all domains of cognition [3]. To date, the only attempt to compare self-processing measures, conducted by Nijhof et al. [19], provided no support for a shared egocentric bias across the attentional and the perceptual domains. Although we did observe a significant correlation between self-biases in these domains, Bayesian statistics indicated only anecdotal evidence in favor of the alternative hypothesis of an association. It should also be noted that the current study adopted a distinct paradigm to assess the self-bias effect in the attentional domain (i.e., the visual search task instead of the attentional blink task), suggesting that the strength of a relationship between different self-bias measures might depend on specific features of the paradigms employed. Furthermore, we found an association between the magnitude of the self-bias in the memory domain and in the attentional domain, while there was no association in self-bias levels between memory and perception domains. Our results are therefore in contrast with a perspective that considers the egocentric bias as a unitary effect, and rather emphasize the heterogeneous nature of the self, which encompasses a multitude of aspects and dimensions that do not necessarily relate to one another.

There are several explanations for the current pattern of results. On the one hand, attentional resources might exert a relevant influence on both the paradigms we employed to address self-reference in the memory and the perceptual domains. In line with this argument, attention may be the common factor driving the self-bias effects on both tasks. On the other hand, rather than affecting cognitive performance on a broader level, one could argue that the nature of the self-specific process underlying the self-bias effects on the different tasks varies. According to previous research, the shape-label matching task would rely on a first-order self-representation [5, 29, 38], involving a ‘subjective’ level of the self, whereas the trait adjectives task would require a second-order self-representation [5, 29, 38], in which the self is the object of participants’ own thought. One could argue that performing the visual search task might also entail higher-order components that are characteristic of second-order self-representations (e.g., explicitly processing self-related stimuli as targets of the search). The fact that both the trait adjectives and the visual search tasks involve features of second-order self-representations might explain the robust correlation we found between the self-bias effects across memory and attention. An alternative explanation could be that the self-bias indices on the trait adjectives and the visual search tasks correlate due to higher verbal demands. Nevertheless, all paradigms under investigation involve some verbal elements, the extent of which varies a lot between the three tasks: while the trait adjective task requires decoding of the semantic meaning of words (e.g., to evaluate how descriptive the word ‘*intelligent’* is, the participant must have knowledge of the semantic meaning of this term), this might not be the case for the visual search task (which only requires detection of a specific name) or for the shape-label matching task (which only requires matching of a specific name). As the self-bias magnitude in the shape-label matching task was unrelated to the size of the self-bias in the trait-adjectives task, and Bayesian analysis provided weak evidence in favor of its correlation with the self-bias in the visual search task, this sets the shape-label matching task apart from the latter two. In this regard, it is worth mentioning that recent findings questioned the nature of the self-bias observed in the shape-label matching task, suggesting that the self-bias effects in this paradigm may not result from the impact of self-reference on perceptual processing, but rather reflect prioritization of self-related items held in working memory [51]. Nevertheless, our overall findings do not support a unitary view of the self-bias effect across distinct tasks/domains of cognition.

The second aim of the current study was to further explore the association between autism-related symptomatology and distinct self-bias measures. Although a number of studies suggested a decreased self-bias as a key feature of individuals with an autism diagnosis [14, 27, 34–37], other studies confuted this hypothesis [19, 29, 38]. However, previous research did not always take into account the type of self-representation (i.e., first- or second-order) specifically deployed in the paradigm under study [29]: while first-order self-referential processing (e.g., labelling an item as ‘self-related’) may be unimpaired in autism, second-order self-referential processing (e.g., evaluating to what extent an adjective is self-descriptive) may be disrupted. Our results, however, do not support any association between self-related processing and autistic traits. It should be noted that although we observed a broad distribution of scores on both measures we used to assess autism-related characteristics (i.e., AQ-10 and SRS-A), the width of such distributions may considerably differ when also including individuals with an autism diagnosis, and further, the possibility of categorical differences between individuals with and without an autism diagnosis cannot be excluded. Therefore, additional studies on self-bias effects in individuals with an autism diagnosis are needed. Nevertheless, our results replicate previous null findings [19, 29, 38] while addressing the potential confound of low statistical power: self-reported autistic traits were not found to significantly correlate with the self-bias magnitude in any of the three domains.

The current study has some limitations. First of all, the entire experimental procedure was completed online. In the last decades, an ever-increasing number of studies has been employing online platforms to investigate human behavior in a convenient and efficient manner [52–54]. Online research may entail certain disadvantages, such as sampling issues (i.e., demographic features of online samples do not always reflect those of offline populations [55]) or systematic ‘self-selection bias’ (i.e., some individuals are more inclined to participate in an online investigation than others [56]). However, online environments have been demonstrated to be suitable experimental settings that consistently produce reliable findings [57]. In agreement with this, a self-bias effect was found across all the three tasks under study, replicating previous investigations conducted in offline settings [10, 14, 24]. Secondly, the present conclusions on the lack of a relation between different measures of self-bias and autistic traits were drawn on the basis of a neurotypical sample. Even though such a dimensional approach has been regarded as an informative methodology to examine autistic characteristics in neurotypicals (e.g. [30]), we cannot exclude the possibility that different findings might be observed in individuals with a formal diagnosis of autism. It may be that actual impairments in self-related processing may emerge in autistic individuals only. Future studies including clinical samples are therefore warranted. Finally, the current study explored the relationship between distinct self-bias measures across cognitive domains uniquely by means of the three experimental paradigms in question. Future research should aim to extend the present evidence by testing a wider set of experimental tasks.

## Conclusions

A robust self-specific cognitive advantage was consistently found when testing participants on three paradigms for measuring self-bias in distinct cognitive domains: the shape-label matching task (perception), the visual search task (attention), and the trait adjectives task (memory). Most importantly, we found that the observed self-bias effects did not always relate to one another, suggesting that the degree of association between distinct self-bias measures varies considerably depending on the domain or the aspect of cognition involved. The present results stand in contrast with a conceptualization of the self-bias as a uniform and unidimensional effect, and rather provide evidence favoring a multifaceted and diversified understanding of the self. The findings of the current investigation, which addressed several methodological limitations of past research, raise awareness on the complexity of self-preferential processing, and extend the current knowledge of how the self affects cognition in both neurotypical and autistic individuals.

## Data Availability

The datasets used and analyzed during the current study are available from the corresponding author on reasonable request.

## Abbreviations

AQ-10: 10-Item Autism Spectrum Quotient
SRS-A: Social Responsiveness Scale—Adult version
SRE: Self-reference effect
RT: Reaction time
SD: Standard deviations
ANOVA: Analysis of variance
BF_10_: Bayes factor in favor of H1 over H0

## Footnotes

1 In the study conducted by Nijhof and colleagues (2020), 54 participants were tested, which corresponds to 80% power for detecting an effect size of d = 0.39 and correlations of r = 0.37 at α = 0.05.
